# Clinical application of functional near-infrared spectroscopy for burn assessment

**DOI:** 10.3389/fbioe.2023.1127563

**Published:** 2023-03-30

**Authors:** Yoo Hwan Kim, Seung-Ho Paik, Youngmin Kim, Jaechul Yoon, Yong Suk Cho, Dohern Kym, Jun Hur, Wook Chun, Beop-Min Kim, Byung-Jo Kim

**Affiliations:** ^1^ Department of Neurology, Hallym University Sacred Heart Hospital, Hallym University College of Medicine, Anyang, Republic of Korea; ^2^ Department of Neurology, Graduate School, Korea University, Seoul, Republic of Korea; ^3^ Department of Bio-convergence Engineering, Korea University College of Health Science, Seoul, Republic of Korea; ^4^ Department of Surgery, Burn and Trauma Center, Daein Surgery and Medical Hospital, Seongnam, Republic of Korea; ^5^ Department of Surgery, Hangang Sacred Heart Hospital, Hallym University College of Medicine, Seoul, Republic of Korea; ^6^ Department of Neurology, Korea University Anam Hospital, Seoul, Republic of Korea; ^7^ BK21 FOUR Program in Learning Health Systems, Korea University, Seoul, Republic of Korea

**Keywords:** burn, near-infrared spectroscopy, oxygen saturation, skin, hemodynamics

## Abstract

**Significance:** Early assessment of local tissue oxygen saturation is essential for clinicians to determine the burn wound severity.

**Background:** We assessed the burn extent and depth in the skin of the extremities using a custom-built 36-channel functional near-infrared spectroscopy system in patients with burns.

**Methods:** A total of nine patients with burns were analyzed in this study. All second-degree burns were categorized as superficial, intermediate, and deep burns; non-burned skin on the burned side; and healthy skin on the contralateral non-burned side. Hemodynamic tissue signals from functional near-infrared spectroscopy attached to the burn site were measured during fNIRS using a blood pressure cuff. A nerve conduction study was conducted to check for nerve damage.

**Results:** All second-degree burns were categorized into superficial, intermediate, and deep burns; non-burned skin on the burned side and healthy skin on the contralateral non-burned side showed a significant difference distinguishable using functional near-infrared spectroscopy. Hemodynamic measurements using functional near-infrared spectroscopy were more consistent with the diagnosis of burns 1 week later than that of the degree of burns diagnosed visually at the time of admission.

**Conclusion:** Functional near-infrared spectroscopy may help with the early judgment of burn extent and depth by reflecting differences in the oxygen saturation levels in the skin.

## 1 Introduction

Clinical assessment of burn severity and prediction of the consequent clinical outcomes are complex and challenging tasks, even for experienced burn specialists. Burn severity depends on the wound depth and affected area (Jeschke et al., 2020). In particular, burn depth is an important factor in evaluating the need for surgical treatment. Although superficial and full-thickness burns are relatively easy to differentiate, unlike other burns, it may be difficult to clearly distinguish between second-degree burns, which can be divided into superficial, intermediate, and deep burns, during the initial evaluation. Clinical assessment based on visual and tactile examinations is a widely used classic method for assessing burn severity. However, the evaluation of burn depth by conventional methods often leads to inaccuracies even when performed by experienced clinicians. The burn specialist’s clinical judgment has been reported with an accuracy of 70%–80% for burn depth assessment (Pape et al., 2001). Decision making with regards to performing surgical interventions such as early excision and grafting and prediction of prognosis are still based on inaccurate methods, therefore more objective quantitative evaluation methods for early burns are needed.

Several optical technologies have been investigated to help clinicians assess burn wound severity (Kaiser et al., 2011; Thatcher et al., 2016b; Lertsakdadet et al., 2022). Burn depth assessment techniques can be divided into two categories such as imaging and non-imaging. Non-imaging methods include clinical judgment, biopsy, and late debridement. Burn imaging devices include color photography, thermography, indocyanine green video angiography, laser doppler, and ultrasonography. A previous animal study showed that these imaging techniques have higher accuracy in imaging depth assessment than clinical judgment alone (Thatcher et al., 2016a). In particular, among various imaging technologies, functional near-infrared spectroscopy (fNIRS) is a promising technique for assessing burn depth (Thatcher et al., 2016b).

fNIRS has often been used to determine regional oxygen saturation in the brain tissue of patients with cerebral ischemia or orthostatic intolerance (Seok et al., 2018; Kim et al., 2020a; Kim et al., 2020b; Phillips et al., 2020). fNIRS can examine a broad range of tissues using light penetration and reflection by light emitter and receiver pairs along with the advantage of being a non-invasive, portable device that can evaluate end-organ perfusion and easily adapted to various environments, including emergency rooms, operating rooms, and hospital wards for bedside monitoring (Jayachandran et al., 2016; Phillips et al., 2023). fNIRS provides surgeons with an early, reliable, and objective indication, allowing for timely surgical intervention (Repez et al., 2008; Whitaker et al., 2012). Our previous study showed the potential of direct cerebral perfusion monitoring using fNIRS during the head-up tilt and Valsalva maneuvers (Kim et al., 2018; Kim et al., 2019). Additionally, we confirmed that adding fNIRS to head-up tilt measurements could better detect cerebral hemodynamic changes in patients with early electrical burns (Kim et al., 2022). However, there are only a few reports of the utility and feasibility of fNIRS in determining the regional tissue oxygen saturation of burn wounds or assessing the burn depth (Anselmo and Zawacki, 1977; Yeong et al., 2005). In recent studies, hyperspectral imaging with wavelengths in the near-infrared range (650–1,050 nm) have shown the potential to discriminate between partial and deep burns in a small pig model, (Calin et al., 2015; Li et al., 2015), however, very few human studies exist in this regard.

We aimed to accurate investigate the differences in hemodynamic changes according to the burn depth between the burn and non-burn sided skin of patients with burns. We hypothesized that fNIRS would accurately determine the burn depth, predict severity, while providing useful references for clinicians for further management.

## 2 Materials and methods

### 2.1 Participants

Patients with burns were recruited from January 2019 to December 2020 based on the following criteria: 1) Second-degree burn injury within 1 week; 2) no history of central or peripheral nervous system diseases; and 3) no history of cardiovascular disease or peripheral artery diseases; and 4) no history of vasopressor or vasodilator administration during the measurement because of their effects on hemodynamics and skin blood flow. 5) Burn injuries in the body trunk were excluded from this study because the fNIRS data can be affected due to values involving the lung and heart injuries; patients with inhalation burn injury who presented with hypoxia, carbon monoxide poisoning, burns with severe pain, or severe burns with poor vital signs that made it difficult to attach the fNIRS probes were excluded. Demographic and clinical data, including age, sex, and comorbid chronic diseases, were also obtained.

### 2.2 Ethical approval

All participants provided written informed consent prior to inclusion in the study. All procedures were performed in accordance with the Declaration of Helsinki and approved by the Hallym University Institutional Review Board (IRB No. 2020-11-015-001). Some figures contain identifiable images of individuals. Written consent for the publication of these images was obtained.

### 2.3 Study design

**FIGURE 1:**
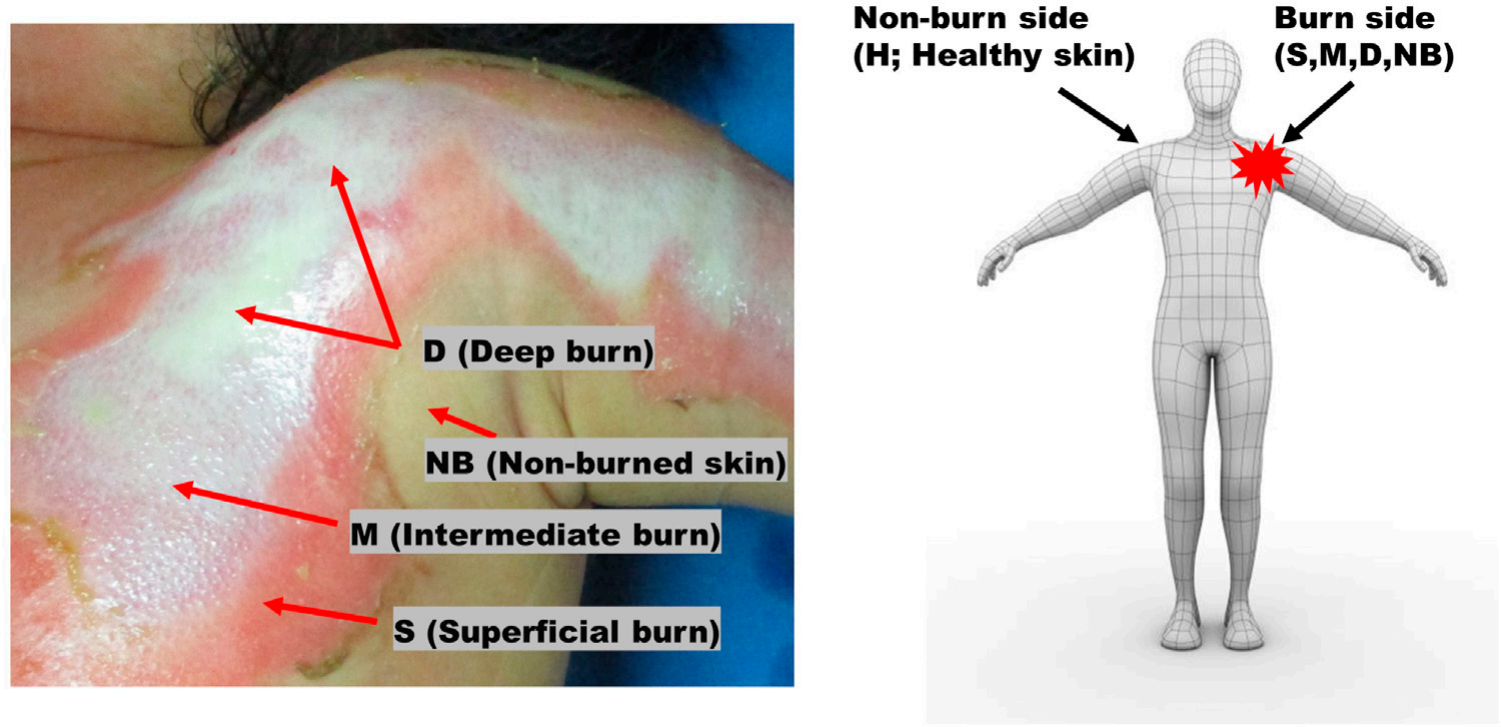
Burns were categorized as superficial (S), intermediate (M), and deep (D) burns, non-burned skin on the burn side (NB), and healthy skin on the contralateral non-burn side (H).

All second-degree burns were categorized into superficial (S), intermediate (M), and deep burns (D); non-burned skin on the burn side (NB); and healthy skin on the contralateral non-burn side (H) (Figure 1). To use the fNIRS technique, a task that causes changes in blood flow is necessary. BP cuff test was used as a task for the purpose of inducing changes in blood flow.

**FIGURE 2:**
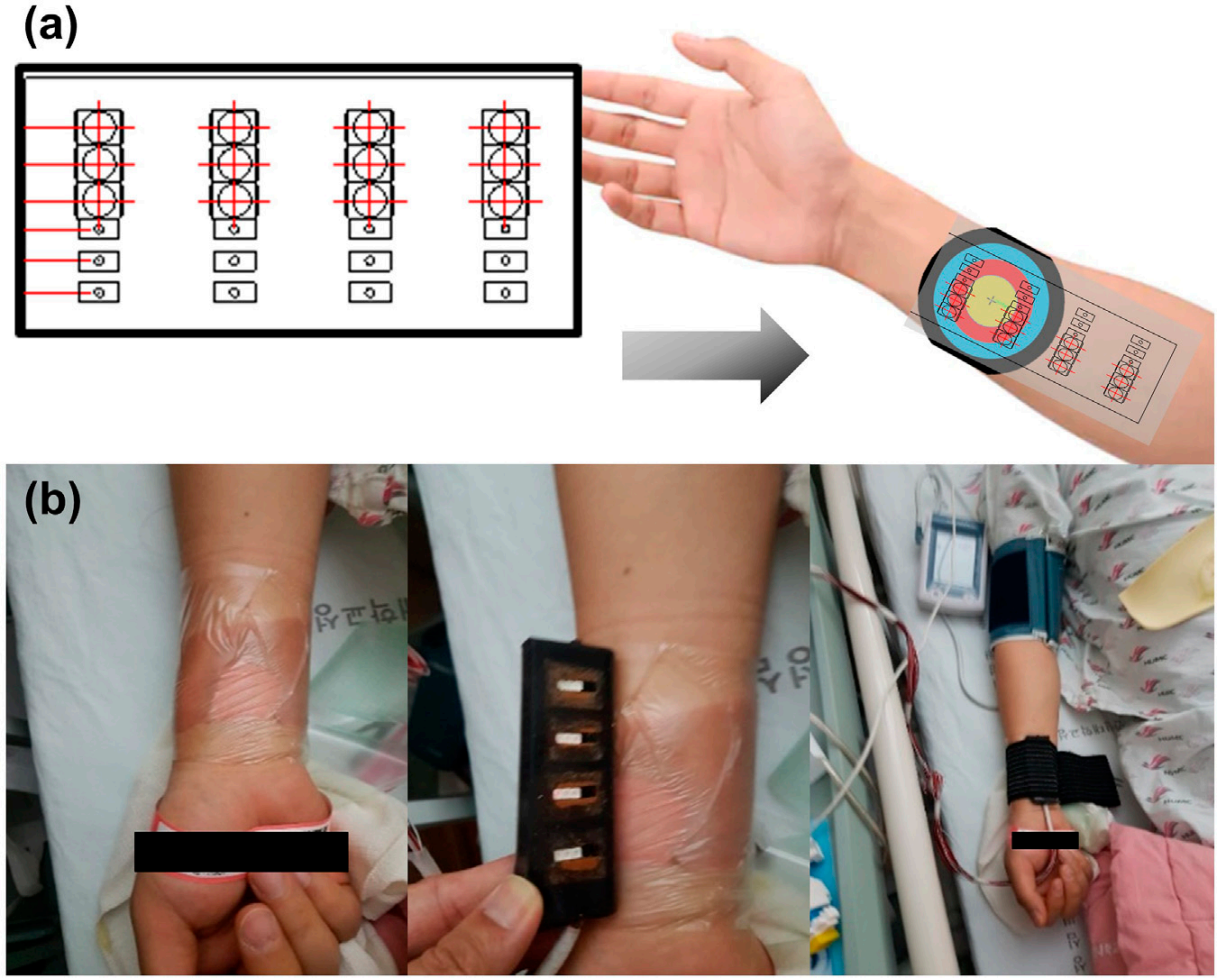
**(A)** Schematic diagram of functional near-infrared spectroscopy (fNIRS) designed to identify the hemodynamic tissue signal at burn sites. **(B)** Actual measurement of the hemodynamic tissue signal using fNIRS attached to the burn site while performing a task using a blood pressure cuff.

The tests were performed in a sequence. When the burn patient was lying down, hemodynamic tissue signals from the fNIRS probes attached to the burn site were measured during fNIRS using a blood pressure (BP) cuff. Patient arms should rest comfortably at heart level. It is important that the cuff fits as a cuff that is too large or too small can change blood pressure readings. A sphygmomanometer cuff was placed over the artery, and serial measurements of BP (systolic and diastolic) and heart rate were obtained. The patients were kept at rest, and the systolic pressure was maintained above 90 mmHg and below 160 mmHg. The systolic and diastolic BPs were displayed on a monitor console. The fNIRS signal was measured on the contralateral side in the same area as the extremity without burns. The surface of the burn wound estimated by fNIRS was covered with a sterile sheet of transparent OPSITE FLEXIFIX Transparent Film (Smith & Nephew, London, United Kingdom) to allow indirect contact between the fNIRS probes and the burn injury. The fNIRS probe and the test site were wrapped with minimal elastic bands to avoid applying pressure to the burned and non-burned skin, so that the fNIRS probe adhered to the skin without any lifting space. By controlling the area where fNIRS is attached to the skin, the blood flow of burned and non-burned skin could be measured simultaneously (Figure 2). A nerve conduction study (NCS) was performed to check for nerve damage. A burn specialist evaluated the burn severity at the time of initial admission and 1 week after admission for preoperative evaluation. The specialist visually classifies the severity of each burn at 4 measurement locations where the fNIRS probe is in contact with the skin. The skin at each of the four measurement locations is classified as one of superficial (S), intermediate (M), and deep burns (D); non-burned skin on the burn side (NB); and healthy skin on the contralateral non-burn side (H). Data were collected according to the type, severity, and burn depth of each patient.

### 2.4 Principles of fNIRS

fNIRS is a non-invasive optical imaging tool for observing hemodynamic changes based on the measurement of oxyhemoglobin (HbO) and deoxyhemoglobin (HbR) concentrations in the blood. fNIRS has been applied extensively in imaging studies on brain activation and living tissues. The physiological activation of neurons leads to an imbalance between oxygen supply and utilization, increasing the concentration of HbO and decreasing the concentration of HbR. Most tissues are relatively transparent to light in the near-infrared range of 700–900 nm, and photons interact with tissues *via* absorption and scattering, indicating that near-infrared light is less absorbed and scattered by tissues than by light at other wavelengths. This range of wavelengths is often called an optical window because light can easily pass through most tissues; however, it is reflected by HbO and HbR, thus, the absorption and scattering of light used for fNIRS can provide information relevant to neural activity. A detailed review of fNIRS principles and terminology can be found in our previous articles (Kim et al., 2018; Kim et al., 2019).

### 2.5 Customized fNIRS device and measures

**FIGURE 3:**
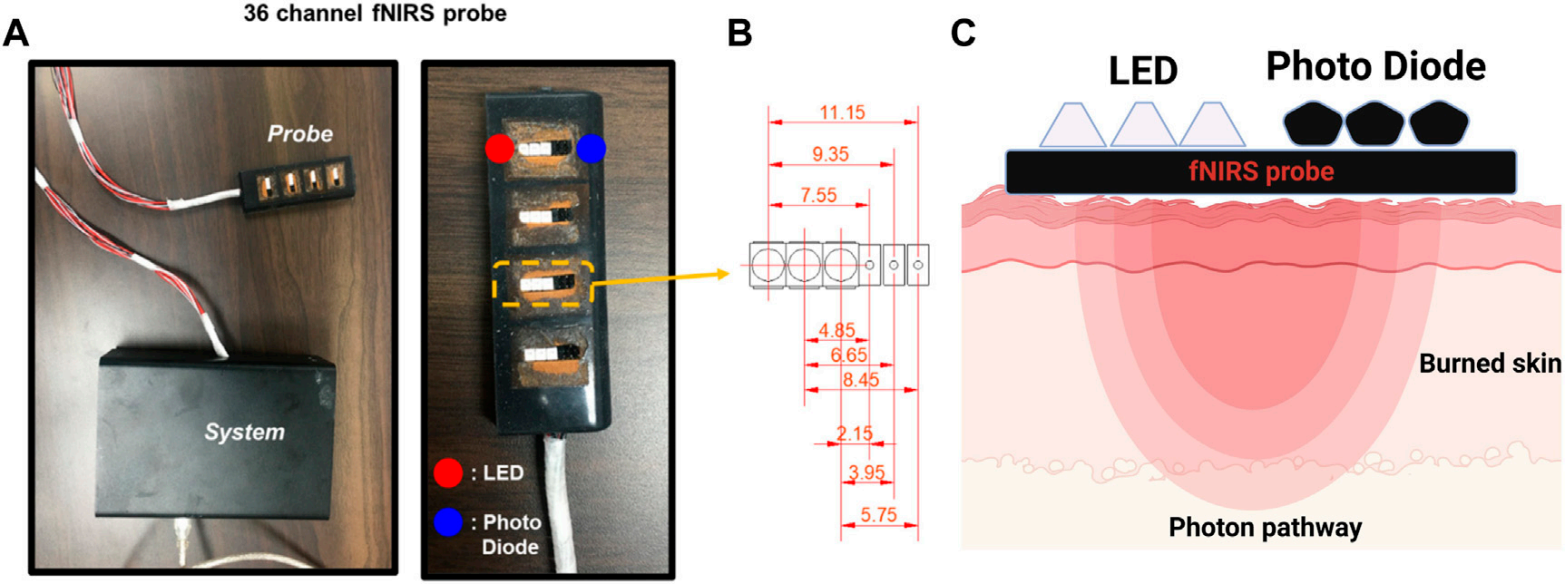
The functional near-infrared spectroscopy (fNIRS) system used in this study. **(A)** Probe and controller used in the clinical experiments. **(B)** The customized 36-channel fNIRS probe consists of 12 LED sources and 12 photodetectors. The figure shows the 9 different source-detector separations: 2.15, 3.95, 4.85, 5.75, 6.65, 7.55, 8.45, 9.35, and 11.15 mm. **(C)** Near-infrared light paths in the skin from the LED to the Photo Diode.

The fNIRS probe developed to measure changes in oxygen saturation during imaging was 25 mm × 75 mm in size and consisted of 36 channels. The probe is made of soft silicone (Ecoflex 00–30, Smooth-On Inc., Macungie, PA, United States) used for special makeup; thus, it does not injure the burned area. It consisted of 12 light sources and 12 photodetectors. The oxygen saturation concentration in the blood was measured using a photodetector (Figure 3A). The distance between the light source and detector was approximately 1–12 mm. The shortest channel measured the signal originating from the epidermis, and the longest channel measured the signal at a depth of approximately 6 mm.

Nine signals were detected at one measurement location. The center distances between channels were 2.15, 3.95, 4.85, 5.75, 6.65, 7.55, 8.45, 9.35, and 11.15 mm (Figure 3B). Because epidermal, dermal, and total skin thicknesses differ according to body site, sex, age, and ethnic origin in healthy body parts, normal values were referred to in the existing literature for skin-related clinical investigations (Oltulu et al., 2018). Based on these reference values, in this study, four layers were composed of channel combinations. The first layer represents hemodynamic information at a depth of 1 mm, reflecting the entire epidermis and some superficial layers of the dermis, averaging the 2.15 mm and 3.95 mm channels. The second layer is an average of the 4.85 mm and 5.75 mm channels, reflecting hemodynamic information at a depth of 1–2 mm corresponding to the intermediate layer of the dermis. The third layer comprises an average of the 6.65 mm and 7.55 mm channels, containing information on the deep dermal layer at a depth of 2–3 mm. The fourth layer is the average of 8.45 mm, 9.35 mm, and 11.15 mm channels, and mainly reflects the information of 3–6 mm depth including the epidermis, entire dermis, and subcutaneous fat layer.

The light source for the fNIRS system uses two wavelengths of light, namely, 760 and 830 nm, and operates at approximately 4 Hz. Unlike normal fNIRS instruments, because the light source and detector are very close, controlling the brightness of the light is crucial. The system uses digital-to-analog converter technology to control the brightness so that the light does not saturate. The operating graphical user interface software based on the MATLAB (The MathWorks, Inc., Natick, MA, United States) program is connected by serial communication to check the signal in real-time and apply a moving average filter to remove real-time noise.

### 2.6 Signal pre-processing and statistical analysis

The signal is the analog to digital converter resolution from 0 to 4,095, measured with a photodetector. The concentration change values of HbO, HbR, and total hemoglobin (HbT) were calculated using the modified Beer–Lambert Law (Villringer and Chance, 1997). The calculated oxygen saturation values were filtered using a first-order band-pass filter (0.01–0.2 Hz) to remove noise (Pinti et al., 2019). In this study, motion noise existed because many patients complained of pain in the burned area. Therefore, principal component analysis was performed to remove the noise with the biggest change, and reconstruction was performed to restore the signal. This technique was applied based on a previous study’s methods (Paik et al., 2019). The analysis figure was smoothed by applying a Gaussian filter to the image area.

The average HbO, HbR, and HbT values for each layer according to the burn degree were analyzed with post-hoc analysis using the Welch test and Dunnett’s T3 correction after analysis of variance was performed. Statistical significance was set at *p* < 0.05, and data are presented as means (range; standard deviation). Statistical analyses were performed using SPSS version 21.0 (IBM Corp., Armonk, NY, United States).

## 3 Results

### 3.1 Participant characteristics

**FIGURE 4:**
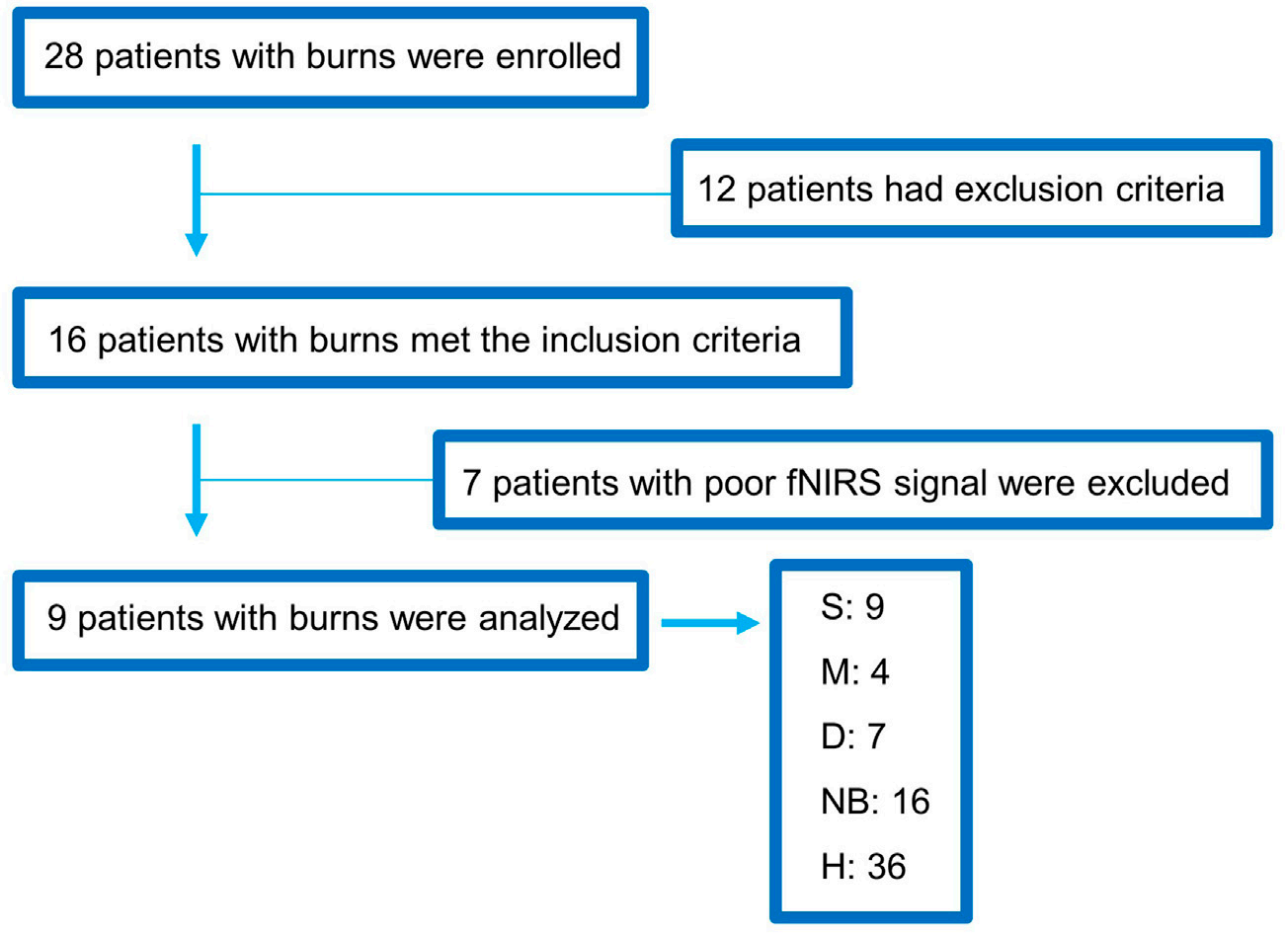
Flowchart showing the enrollment of the study population. fNIRS, functional near-infrared spectroscopy; S, superficial burn; M, intermediate burn; D, deep burn; NB, non-burned skin on the burn side; H, healthy skin on the contralateral non-burn side.

A total of 28 patients with burns were enrolled in this study, of which 12 were excluded from the analyses because they had severe arrhythmia, inhalation burns, or central and peripheral nerve diseases. Additionally, seven patients were excluded because of excessive motion artifacts and poor fNIRS signals during the BP measurement. Finally, data from nine patients with burns were analyzed. Among the nine patients, the burn side can be classified as a total of 36 burns, considering 4 measurement locations per patient, and healthy skin on the contralateral non-burn side (H) also totaled to 36. All analyzed patients had second-degree burns, including S (9), M (4), D (7), NB (16), and H (36) (Figure 4). Five of the nine patients were men, and the most common burn types were scalding and flame burns, followed by chemical burns. A NCS was performed at the burn site, and the F-wave results were obtained up to 440 times (Table 1).

**TABLE 1:** Demographic and clinical characteristics of the participants.

Case	1	2	3	4	5	6	7	8	9
Sex	F	M	M	M	M	F	F	M	F
Age	36	30	25	22	27	41	72	20	54
Height m)	1.58	1.78	1.78	1.76	1.63	1.63	1.5	1.74	1.6
Weight (kg)	80	78	84	87	38	51	64	65	47
BMI (kg/m^2^)	32.05	24.62	26.51	28.09	14.3	19.2	28.44	21.47	18.36
Burn	chemical	flame	scalding	flame	flame	flame	scalding	scalding	scalding
TBSA (%)	14	4	4	14	70	25	20	5	4
Burn area	Lt elbow	Lt wrist	Rt ankle	Rt forearm	Rt forearm	Rt hand	Rt elbow	Rt ankle	Lt foot
NCS	—	N-S	—	Rt ulnar N	Rt median, ulnar, radial N	Rt median, ulnar, radial N	—	N-S	Lt superficial peroneal N
F-wave (440 times)	—	Rt median 26.93	—	Rt median 26.82	Rt median NR	Rt median 26.82	—	Rt peroneal 46.04	Rt peroneal 42.24
		Rt ulnar 27.34		Rt ulnar 27.71	Rt ulnar NR	Rt ulnar 24.64		Rt tibial 43.65	Rt tibial 45.52
		Lt median 27.03		Lt median 26.46	Lt median NR	Lt median 27.86			Lt peroneal 43.65
		Lt ulnar 24.22		Lt ulnar 27.40	Lt ulnar 31.15	Lt ulnar 24.53			Lt tibial 46.30

BMI, body mass index; TBSA, total burn surface area; NCS, nerve conduction study; Lt, left; Rt, right; N-S, no specific findings; N, neuropathy; NR, no response.

### 3.2 fNIRS responses to skin burns

**FIGURE 5:**
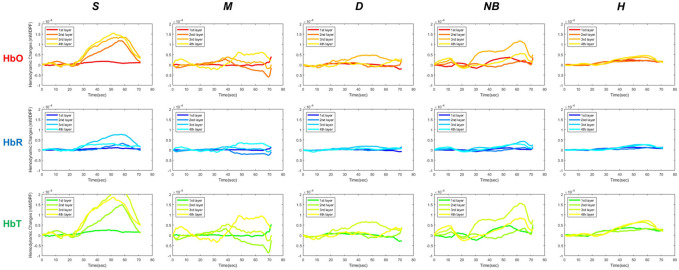
Hemodynamic differences in functional near-infrared spectroscopy in S, M, D, NB, and H. S, superficial burn; M, intermediate burn; D, deep burn; NB, non-burned skin on the burn side; H, healthy skin on the contralateral non-burn side; HbO, oxyhemoglobin; HbR, deoxyhemoglobin; HbT, total hemoglobin.

Changes in HbO, HbR, and HbT over time were analyzed to assess the hemodynamic differences in S, M, D, NB, and H by using fNIRS (Figure 5). As pressure was applied using the BP cuff, it was confirmed that the change in the hemodynamic tissue signal of fNIRS attached to the burn site occurred gradually from 20 s. In the S group, the HbO concentration in the surface layer (first layer) was lower than that in the intermediate (second layer) and deep layers (third and fourth layer). However, in the D group, the overall concentrations of HbO and HbT declined in the surface layer (first layer) and the intermediate layer (second layer), and the concentration in the deep layer (third layer) was relatively maintained. In the NB group, blood flow fluctuated considerably in each layer with time, but in the H group, the fluctuation range was small and stable.

**FIGURE 6:**
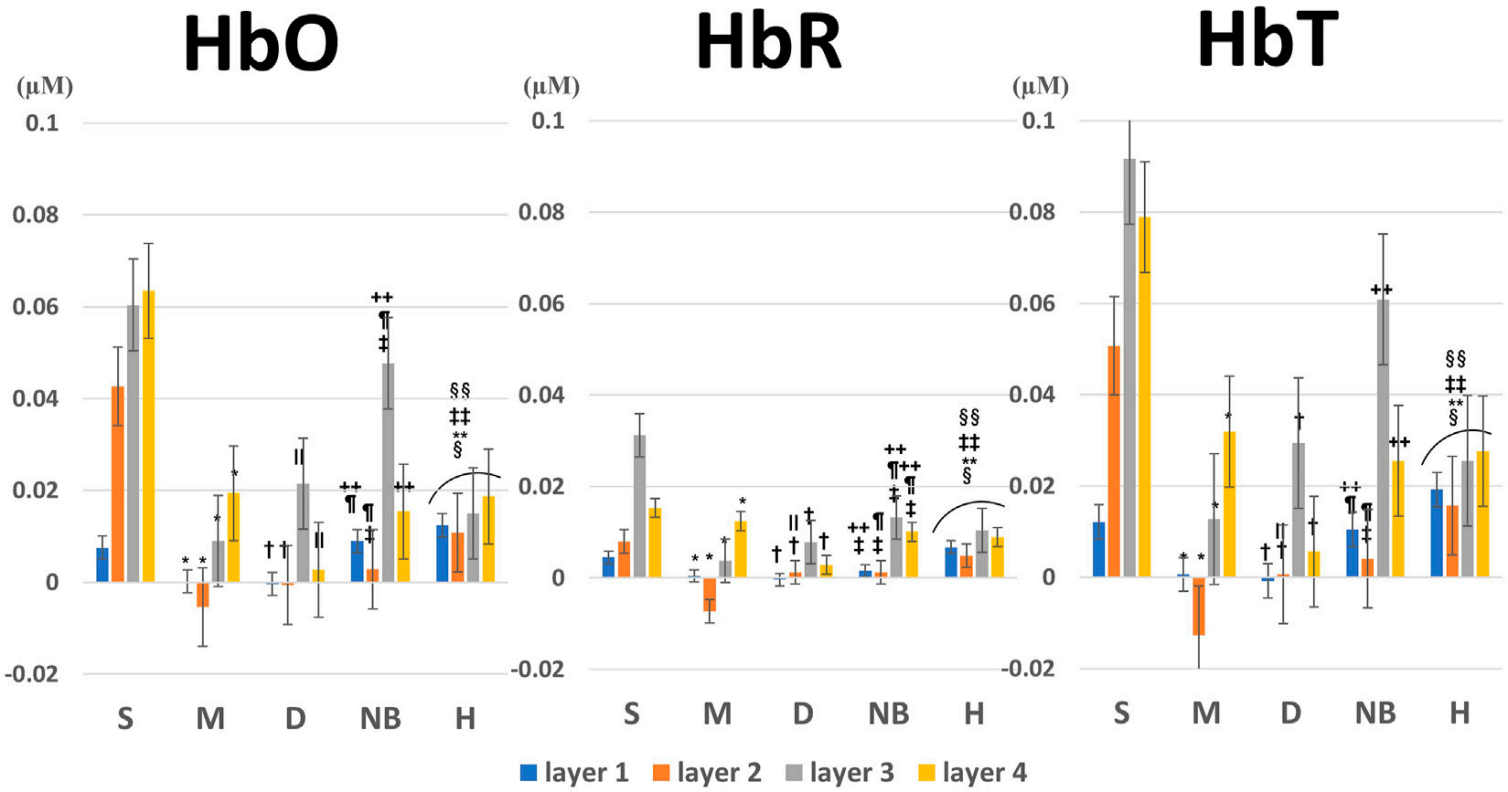
Average and variance of HbO, HbR, and HbT values for each layer according to burn degree. *p*-values were obtained by analysis of variance. The footnote markers indicate statistical significance for post-hoc analysis by the Welch test with Dunnett’s T3 correction as follows: *: S versus M, †: S versus D, ‡: S versus NB, §: S versus H, ∥: M versus D, ¶: M versus NB, **: M versus H, ++: D versus NB, ‡‡: D versus H, §§: NB versus H. HbO, oxyhemoglobin; HbR, deoxyhemoglobin; HbT, total hemoglobin; S, superficial burn; M, intermediate burn; D, deep burn; NB, non-burned skin on the burn side; H, healthy skin on the contralateral non-burn side.

**FIGURE 7:**
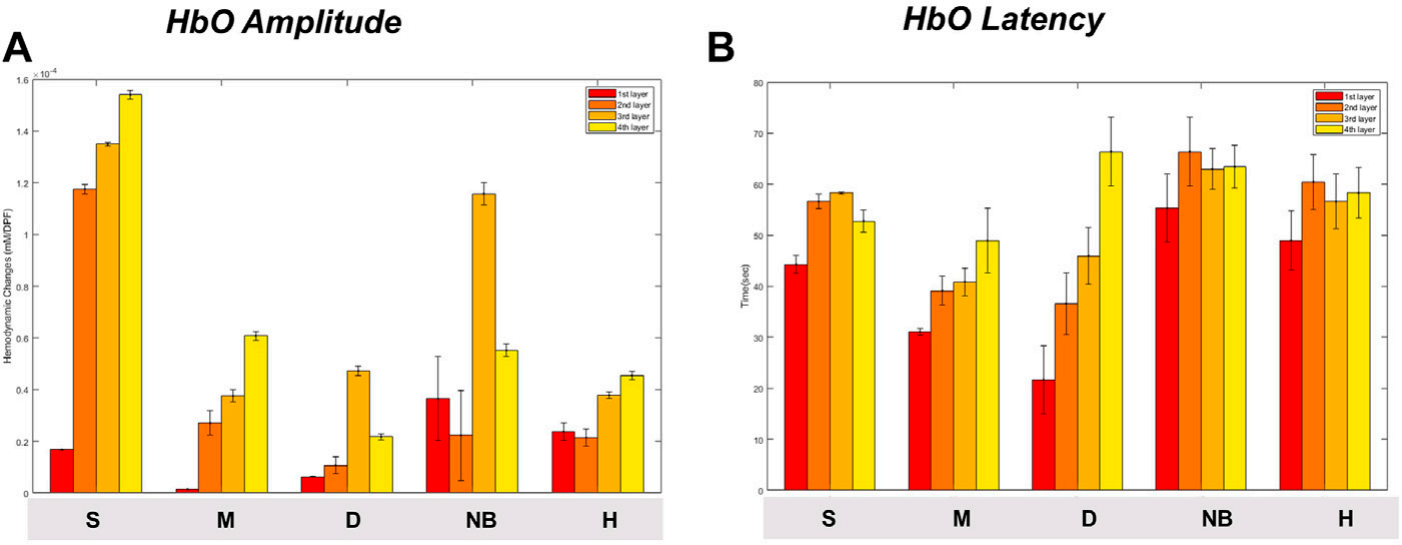
Maximum oxyhemoglobin value [**(A)** amplitude, **(B)** latency] and variance for each degree of burn according to burn depth. Data are presented as means. S, superficial burn; M, intermediate burn; D, deep burn; NB, non-burned skin on the burn side; H, healthy skin on the contralateral non-burn side.

When comparing the average HbO, HbR, and HbT values of each layer according to the burn degree, a statistically significant difference was in general according to the burn degree for each layer (Figure 6). Figure 5 is a graph showing the average values for each subgroup of S, M, D, NB, and H. The maximum value among several peaks of each curve was expressed as an amplitude, and the latency at that point was calculated and shown in Figure 7. It was expressed as HbO amplitude in Figure 7A, and the latency of the maximum concentration values are shown as HbO latency in Figure 7B. In Figure 7, when the amplitude and latency of the point where the maximum HbO value was measured in each of the 4 layers for each burn were graphed, there was no difference in the latency between the groups. However, there was a difference in amplitude. The blood flow in the entire layer gradually decreased from the S group to the D group. The H group maintained a relatively constant blood flow for each layer, whereas the NB group exhibited large fluctuations in each layer.

### 3.3 Case results

**FIGURE 8:**
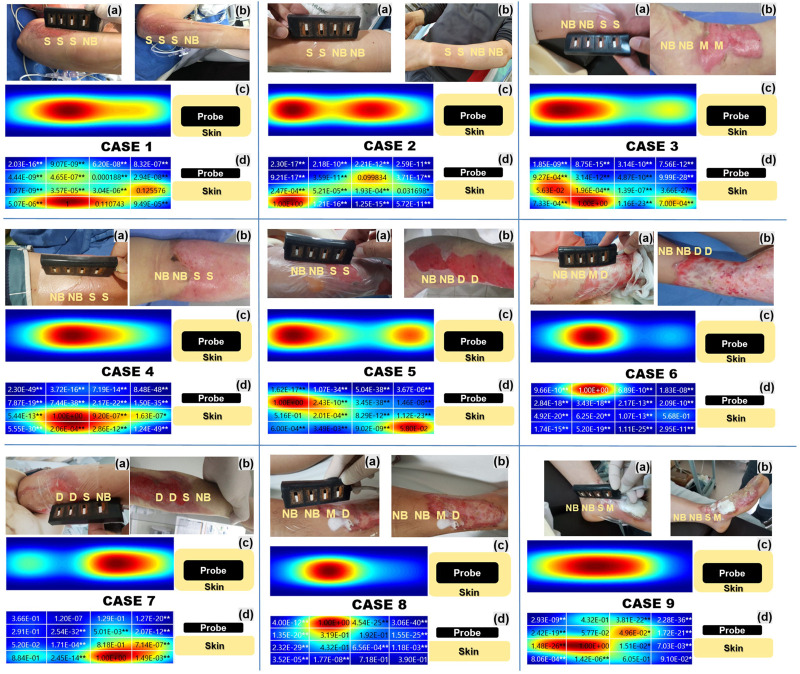
Intuitive functional near-infrared spectroscopy (fNIRS) image mapping and nerve conduction studies of all nine cases indicating the depth and extent of the burns. **(A)** Shows the burn degree on the first day of admission, and **(B)** shows the burn degree after 1 week **(C)** Intuitive fNIRS oxyhemoglobin mapping seen from above. **(D)** Intuitive fNIRS oxyhemoglobin mapping viewed from the side is divided into 16 regions according to the depth and extent of the burn and statistical significance with the adjacent regions. The Wilcoxon rank-sum test was used to perform the statistical analysis. *p < 0.05, **p < 0.01.

Figure 8A shows the burn site at the time of the first visit in all nine cases, and Figure 8B shows the same site 1 week later. Figures 8C, D shows intuitive fNIRS image mapping indicating the depth and degree of burns in the nine cases enrolled in this study, and the statistical significance for each area. Intuitive fNIRS image mapping was divided into 16 regions according to the depth and extent of the image, and statistical significance was observed for each surrounding area based on the part with the highest HbO value. An NCS was performed, except for patients with severe burns or vital signs that were difficult to test.

#### 3.3.1 Case 1

Burn severity in Case 1, assessed twice by burn specialists, was consistent (a: S/S/S/NB, b: S/S/S/NB). In the NB, more activated blood flow layers were formed on the surface than in the S. It was a burn of the left ulnar nerve area at the elbow, however, an NCS was not performed.

#### 3.3.2 Case 2

Burn severity in Case 2, assessed twice by burn specialists, was consistent (a: S/S/NB/NB, b: S/S/NB/NB). S and NB showed a distinguishable HbO difference, and similarly to Case 1, the HbO concentration in surface layers 1 and 2 was lower in S than in NB. It was a burn of the radial nerve area on the left wrist, however, the NCS showed no specific findings in the burn area.

#### 3.3.3 Case 3

The burn severity of Case 3 was evaluated by changing the area that was judged to correspond to S at the first visit to M after a week (a: NB/NB/S/S, b: NB/NB/M/M). Unlike the NB, the HbO concentration in the M was hardly activated except for in the deepest layer. It was a burn of the right tibial area at the ankle, but an NCS wasn’t performed.

#### 3.3.4 Case 4

Burn severity in Case 4, assessed twice by burn specialists, was consistent (a: NB/NB/S/S, b: NB/NB/S/S). Unlike other cases, no typical pattern could explain the correlation between burn severity and HbO concentration. The NCS results were consistent with those of right ulnar neuropathy and showed that the amplitude was decreased and latency delayed in the compound muscle action potential of the right ulnar nerve, which was the burn site.

#### 3.3.5 Case 5

As in Case 3, the burn severity of Case 5 was evaluated by changing the area that was judged to correspond to S at the first visit to D after a week (a: NB/NB/S/S, b: NB/NB/D/D). The D region showed a marked reduction in HbO concentration in the superficial and intermittent layers compared to that in the deep layer. The NCS results showed decreased amplitude and delayed latency of the compound muscle action potential and sensory nerve action potential of the right median, ulnar, and radial nerves at the elbow within the burn site.

#### 3.3.6 Case 6

The burn severity of Case 6 was evaluated by changing the area that was judged to correspond to M at the first visit to D 1 week later (a: NB/NB/M/D, b: NB/NB/D/D). NB showed an activated HbO concentration in the surface layer, but on the contrary, almost no HbO concentration was observed in the entire layer of D. NCS results showed decreased amplitude and delayed latency of the compound muscle action potential of the right median, ulnar, and radial nerves at the wrist within the burn site.

#### 3.3.7 Case 7

The burn severity in Case 7, assessed twice by burn specialists, was consistent (a: D/D/S/NB; b: D/D/S/NB). HbO concentration was barely activated in D compared to S. It was a burn of the right ulnar nerve area at the elbow, however, an NCS was not been performed.

#### 3.3.8 Case 8

The burn severity in Case 8, assessed twice by burn specialists, was consistent (a: NB/NB/M/D, b: NB/NB/M/D). M and D showed similar HbO concentrations, but surface blood flow was more activated in M than in D. It was a burn of the right saphenous nerve area, but the NCS showed no specific findings in the burn area.

#### 3.3.9 Case 9

Burn severity in Case 9, assessed twice by burn specialists, was consistent (a: NB/NB/S/M; b: NB/NB/S/M). The surface HbO concentration was lower in NB than in S, contrary to our expectations. The HbO concentration of the entire layer of D was hardly activated. The NCS results showed decreased amplitude and delayed sensory nerve action potential in the left superficial peroneal nerve at the foot within the burn site.

In Cases 3, 5, and 6, the severity of the burns changed when re-evaluated by the burn specialist 1 week later. As can be seen in these cases, fNIRS values measured at the first admission showed a pattern that was consistent with the degree of change in burn severities reconfirmed 1 week later. Except for Cases 4 and 9, blood flow in the superficial layer of the NB was good compared to that observed in other burn severities. In intuitive fNIRS oxyhemoglobin mapping, the blood flow classification of burn severity is generally clearer when the blood flow is compared with that in surrounding NBs rather than independent discrimination. The blood flow of S was formed relatively clearly in the deep layer, but there was a difference between M and D as blood flow was hardly formed. However, it was difficult to distinguish whether the part was M or D. Even if they were classified as the same NB, there was a difference in the blood flow of the NB in each case.

## 4 Discussion

Depending on the severity of the burn, each patient’s burned skin differed significantly in terms of blood oxygenation levels determined using fNIRS. In addition, fNIRS use confirmed that the normal appearing skin around the burn site was hemodynamically different from the skin on the non-burned side. Hemodynamic measurements using fNIRS were more accurate than the visual diagnosis of the burn degree at the time of admission. This suggests that fNIRS may help determine the extent and depth of burns in the early stages of injury by reflecting differences in oxygen saturation in the burn areas.

These aspects are to be remembered when evaluating patients, as the depth of the burn determines the treatment plan. First-degree and third-degree burns are relatively easy to identify at the time of presentation. Unlike other burns, second-degree burns may have subtle differences in the severity of the burn at initial evaluation. Second-degree burns can be divided into superficial, intermediate, and deep injuries. Superficial wounds heal within 3 weeks, whereas deep wounds above the intermediate layer may take longer to heal or require excision and grafting; thus, evaluating the depth of the burn early is important in the case of second-degree burns.

In second-degree superficial burns, the HbO concentration in the superficial layer was relatively low compared to other layers, but the blood flow in the entire layer was maintained to some extent. Deeper burns are correlated with a lower HbO concentration, not only in the surface layer, but also in the deep part of the middle layer, while the blood flow throughout the layer also decreases. Although the skin may appear normal, the skin near the burn site was hemodynamically different from the area without burns. Difference in blood flow distribution in each of the nine cases with the same NB is thought to be due to the blood flow in the NB being affected by the hemodynamic compensatory mechanism according to the degree of burns around the NB. We presented the possibility of early identification of superficial, intermediate, and deep second-degree burns using fNIRS. Early detection of the severity and depth of burns using fNIRS facilitates decision-making regarding early treatment and helps predict prognosis (Wang et al., 2019).

Burn injuries can be divided into three zones (Herndon, 2018), which represent a cellular response at the border between the most severely damaged skin and the surrounding healthy skin (Thatcher et al., 2016b). The coagulation zone is the point most damaged with irreversible tissue damage. Surrounding this zone is the zone of stasis, an area of the wound that can potentially necrose with inadequate treatment or heal with adequate perfusion. If the patient is under-resuscitated, this burn site may become part of the coagulation zone (Hinshaw, 1963). The third zone, at the edge of the burn injury, is the zone of hyperemia, which is likely to heal with treatment if perfusion is maintained and there is no infection (Hettiaratchy and Dziewulski, 2004; Papp et al., 2004). The fNIRS probe used in our study was designed to better detect burn injuries in these three zones. The intuitive fNIRS image mapping technique indicating the depth and extent of burns of the nine cases facilitated easy recognition of hemodynamics andrevealed the statistical significance for each area clearly.

The degree of burns diagnosed visually at the time of admission and degree of burns diagnosed again after 1 week differed in approximately 33% (3/9) cases. The burn severity of Case 3 was evaluated by changing the area judged to correspond to S at the first visit to M later. In Case 5 the lesion considered S at the first visit changed to D after 1 week, indicatingthat the lesion initially evaluated as S was close to the zone of stasis, and the lesion may have worsened due to lack of appropriate treatment. The burn severity of Case 6 was evaluated by changing the area that was judged to correspond to M at the first visit to D later. In Case 6, only M adjacent to NB was changed to D. Based on the severity of burns measured on the day of admission, D was considered a zone of coagulation, M a zone of stasis, and NB close to the zone of hyperemia. Conversion of burned skin often results from hypoperfusion, dry wounds, swelling, and infection. It is important to identify the zone of stasis early and establish an appropriate treatment strategy when treating burns. In cases 3, 5, and 6, the blood flow of fNIRS was worse than that observed during the initial visual judgment; thus, better treatment results could have been obtained if active treatment, including surgical treatment, was implemented earlier than observation for 1 week.

In this study, flow fluctuations with a duration of about 10 s were generally observed during fNIRS measurements. A Mayer wave is a wave caused by periodic changes or oscillations in arterial blood pressure, which occurs during continuous blood pressure measurements and has a frequency of about 0.1 Hz. It can be defined as an arterial blood pressure oscillation with a frequency slower than the respiratory frequency and is an index showing the strongest and most important coherence with sympathetic nerve activity. To remove the noise of the signal, low pass and band pass filters were used, but high frequency (device and electrical noise) and very low frequency (signal draft) noises were also removed, and biosignals were secured as much as possible. In this way, the Mayer wave frequency becomes more prominent, though a filtering method to improve it is sought. This is our first study that uses fNIRS to assess the difference between burned skin and non-burned skin. The preprocessing settings will be improved for further studies.

This study has several limitations. First, the sample size was small because this was a study of the customized application of fNIRS in patients with burns. Only tests performed on the extremities were included in this study. Given the condition of the patient and the purpose of the study, only second-degree burns were included. Patients with third-degree burns were excluded due to poor vital signs and deep burns, and applying the current fNIRS equipment was difficult in these cases. First-degree burn patients were also excluded because the epidermis was too thin, making accurate measurement and analysis difficult with the current fNIRS equipment. In particular, the number of analyzed cases was greatly reduced by excluding cases where fNIRS attachment was difficult, such as cases with joints or a large amount of signal noise. Second, considering the thin skin of burn patients, a more detailed data analysis would have been possible if the fNIRS device channel had been configured more tightly. Second-degree burns often present at a skin depth of 0.12–2.0 mm. (Wang, 2009). The custom 36-channel fNIRS probe consists of 12 light emitting diode sources and 12 photodetectors that try to detect subtle depth differences, but with obvious limitations. Finally, an NCS was performed to check for nerve damage, however, varying patient’s condition limited the wide application of NCS and interpretation of burn neuropathy. fNIRS may help with the early assessment of burn extent and depth by reflecting the differences in oxygen saturation levels in an affected burn site. This can change the traditional way of assessing burns visually alone and can help to accurately assess the severity and depth of burns early and plan treatment accordingly.

## Data Availability

The original contributions presented in the study are included in the article/supplementary material, further inquiries can be directed to the corresponding author.
